# α-Klotho levels in girls with central precocious puberty: potential as a diagnostic and monitoring marker

**DOI:** 10.3389/fendo.2024.1383812

**Published:** 2024-04-08

**Authors:** Jun-Hong Park, Eu-Seon Noh, Il Tae Hwang

**Affiliations:** ^1^ Department of Pediatrics, Hallym University College of Medicine, Chuncheon, Republic of Korea; ^2^ Department of Pediatrics, Kangdong Sacred Heart Hospital, Seoul, Republic of Korea

**Keywords:** α-Klotho, IGF-1, central precocious puberty, GnRHa, puberty

## Abstract

**Background:**

Recent studies suggest a link between the Klotho protein, sex hormones, and insulin-like growth factor-1 (IGF-1), indicating that α-Klotho levels may rise during puberty, including in central precocious puberty (CPP) cases. This study aimed to explore α-Klotho levels in girls with CPP to assess its potential as a diagnostic and monitoring tool for this condition.

**Methods:**

In total, 139 girls, comprising 82 patients diagnosed with CPP and 57 healthy prepubertal controls, were enrolled in this study. From March 2020 to May 2023, we assessed both α-Klotho levels and clinical parameters. α-Klotho concentrations were measured using an α-Klotho ELISA kit. For the girls with CPP, we additionally analyzed samples taken 6 months after GnRH agonist treatment.

**Results:**

α-Klotho levels were higher in the CPP group compared with the control (CPP group: 2529 ± 999 ng/mL; control group: 1802 ± 675 pg/mL) (P < 0.001), and its level modest decreased after 6 months of GnRH agonist treatment (2147± 789 pg/mL) (P < 0.001). The association between α-Klotho and IGF-1 SDS, follicular stimulating hormone and baseline luteinizing hormone was assessed by partial correlation after adjusting for age, BMI SDS (r= 0.416, p= <0.001; r= 0.261, p= 0.005; r= 0.278, p= 0.002), respectively. Receiver operating characteristic curve analysis identified an α-Klotho cut-off differentiating CPP from controls, with a cut-off of 1914 pg/mL distinguishing girls with CPP from controls with a sensitivity of 69.5% and specificity of 70.2%; the area under the curve was 0.723.

**Conclusion:**

The findings of our study are the first step towards deciphering the role of α-Klotho in puberty induction. With additional data and further research, α-Klotho could potentially be utilized as a significant diagnostic and monitoring tool for CPP.

## 1 Introduction

Central precocious puberty (CPP) is a relatively uncommon but significant pediatric endocrine disorder characterized by premature activation of the hypothalamic-pituitary-gonadal axis, resulting in the early onset of pubertal development, typically before the age of 8 years in girls (1). CPP not only leads to physical and psychological challenges for affected children and their families but also necessitates early diagnosis and appropriate management to mitigate potential long-term health consequences (2). Although tools for CPP diagnosis have been established (3, 4), continuous research is being conducted on new markers to improve diagnosis and monitoring (5–7). Understanding the causes and progression of CPP can aid the development of enhanced diagnostic tools and contribute to the advancement of improved treatment options.

The Klotho protein is encoded by the *Klotho* gene and exists in multiple forms, including transmembrane and soluble forms. The soluble form of Klotho protein is released into the circulation and exerts endocrine functions (8). Notably, Klotho is predominantly expressed in the hypothalamus-pituitary-ovary axis, which is associated with reproductive endocrine-related diseases (9). In line with this, previous studies have shown that in mice, Klotho is moderately expressed in ovarian and pituitary tissues, with a positive correlation with basal LH levels (10). Furthermore, a 2007 study on female mice reported a potential relationship between estradiol and Klotho (11), and a study conducted in 2022 confirmed a connection between α-Klotho and sex hormones among American male participants (12). Recent research has also shown a strong relationship between the Klotho protein and the GH/insulin-like growth factor-1 (IGF-1) system (13). It can be postulated that the levels of sex hormones, IGF-1, and Klotho are organically interconnected. Based on these studies, we hypothesize that α-Klotho may provide valuable insights into the pathophysiology of CPP, its progression, and the effectiveness of therapeutic interventions. This study aimed to investigate the levels of α-Klotho in girls diagnosed with CPP and evaluate its utility as a diagnostic and monitoring marker.

## 2 Materials and methods

### 2.1 Study population

This study included 139 girls, comprising 82 girls diagnosed with CPP and 57 healthy prepubertal control girls. Patients with a known brain tumor, those exposed to cranial irradiation, and those presenting central nervous system-related symptoms were excluded from the study. Patients diagnosed with CPP were treated with subcutaneous administration of leuprorelin acetate (1.875 mg (0.5 vial) for <20 kg, 2.81 mg (0.75 vial) for 20-30 kg, 3.75 mg (1 vial) for ≥30 kg) every 4 weeks. Medical records were collected for both groups, including data before CPP diagnosis and 6 months after the initiation of GnRH agonist treatment. This study was approved by the Institutional Review Board of Hallym University Kangdong Sacred Heart Hospital (Institutional Review Board No. 2020-05-010-009).

### 2.2 Definition of CPP

The criteria for diagnosing CPP in this study required the fulfillment of all three of the following: breast development occurring before the age of 8 years. Bone age (BA) was advanced by 1 year compared to chronological age (CA). A peak luteinizing hormone (LH) concentration of ≥ 5 IU/L during the GnRH stimulation test.

### 2.3 Measurements

The following laboratory parameters were measured in the study participants: LH, follicular stimulating hormone (FSH), Estradiol, IGF-1, alkaline phosphatase (ALP), calcium, phosphate, α-Klotho. Serum levels of α-Klotho were quantified using an α-Klotho enzyme-linked immunosorbent assay (ELISA) kit from Immuno-Biological Laboratories Co., Japan.

### 2.4 Statistical analysis

Statistical analyses were performed using SPSS version 21.0. To compare continuous variables between groups (control, before, and 6 months after GnRH agonist treatment), the t-test was used for independent and paired samples. Pearson’s correlation analysis was conducted to explore the relationship between α-Klotho levels and other clinical variables.

Multivariable logistic regression analysis was performed to investigate the association between serum α-Klotho levels and CPP diagnosis. Receiver operating characteristic (ROC) curves were constructed to assess the diagnostic performance of serum α-Klotho levels in distinguishing prepubertal control girls from girls with CPP.

## 3 Results

The clinical, hormonal, and metabolic parameters of controls and girls with CPP at diagnosis are presented as mean ± standard deviation values. Girls with CPP exhibited significantly higher levels of LH, estradiol, ALP, and IGF-1 SDS than control girls (**Table 1**). The α-Klotho levels in control girls were significantly lower (mean: 1802 pg/mL) than those in girls diagnosed with CPP (mean: 2529 pg/mL), showing a statistically significant difference (p = 0.001).

**Table 1:** Clinical, hormonal, and metabolic parameters of controls and girls with CPP at Diagnosis.

	Control (Prepubertal) N = 57	CPP N = 82	P-value
**Age (years)**	8.39 ± 0.81	8.42 ± 0.65	NS
**BMI SDS**	-0.29 ± 0.88	0.29 ± 1.17	0.001
**Basal LH (mIU/mL)**	0.26 ± 0.08	0.60 ± 0.59	<0.001
**Basal FSH (mIU/mL)**	2.27 ± 1.79	2.96 ± 1.49	NS
**Peak LH (mIU/mL)**	Not Done	12.88 ± 8.65	Not Comparable
**Peak FSH (mIU/mL)**	Not Done	11.50 ± 3.72	Not Comparable
**Estradiol (pg/mL)**	5.6 ± 2.6	12.1 ± 11.9	<0.001
**Calcium (mg/dL)**	9.85 ± 0.25	9.74 ± 0.67	NS
**Phosphate (mg/dL)**	4.85 ± 0.53	4.89 ± 0.46	NS
**ALP (IU/L)**	237.7 ± 49.1	342 ± 7.65	<0.001
**IGF-I SDS**	-0.39 ± 0.21	0.004 ± 0.26	<0.001
**α-Klotho (pg/mL)**	1802.72 ± 675.68	2529.36 ± 999.51	<0.001

BMI, body mass index; CPP, central precocious puberty; N, number; NS, not significant; SDS, standard deviation score.

Partial correlation analysis adjusted for age and BMI (body mass index) SDS was performed to investigate the relationship between α-Klotho and other clinical variables (**Table 2**). Results demonstrated that α-Klotho had a significant positive correlation with basal LH, basal FSH, and estradiol. Notably, IGF-1 SDS displayed a stronger correlation, with a coefficient of 0.416, which was higher than that of the other variables.

**Table 2:** α-Klotho relationship with LH, FSH, estradiol, and IGF-I SDS studied by partial correlation with adjustment for age and BMI SDS.

	Total	
	r	P
**Basal LH**	0.278	0.002
**Basal FSH**	0.261	0.005
**Estradiol**	0.066	0.478
**IGF_1 SDS**	0.416	<0.001

SDS, standard deviation score.

A multivariable logistic regression analysis was performed to assess the impact of α-Klotho on the diagnosis of CPP (**Table 3**). The basal LH, estradiol, Klotho variables were used in their log-transformed and standardized forms. In the first step, controlling for age and BMI, α-Klotho exhibited a statistically significant association with CPP diagnosis. In the second step, adding basal LH, estradiol, and α-Klotho as variables, α-Klotho remained significantly associated with CPP diagnosis (odds ratio: 2.24, p < 0.05).

**Table 3:** Multivariable logistic regression analysis results for CPP.

Step	Variable	B	SE	Wald	p	Odds
1	**Age**	0.50	0.37	1.79	0.181	1.65
	**BMI**	0.38	0.25	2.30	0.129	1.46
2	**Basal LH**	2.94	1.20	6.05	0.014	18.90
	**Basal FSH**	-0.11	0.17	0.41	0.520	0.90
	**Estradiol**	1.02	0.38	7.30	0.007	2.76
	**α-Klotho**	0.81	0.31	6.92	0.009	2.24
-2LL=92.27, Pseudo R^2^ = 0.523						

The Basal LH, Estradiol, Klotho variables were log-transformed and standardized.

BMI, body mass index; CPP, central precocious puberty; SDS, standard deviation score.

**Figure 1:**
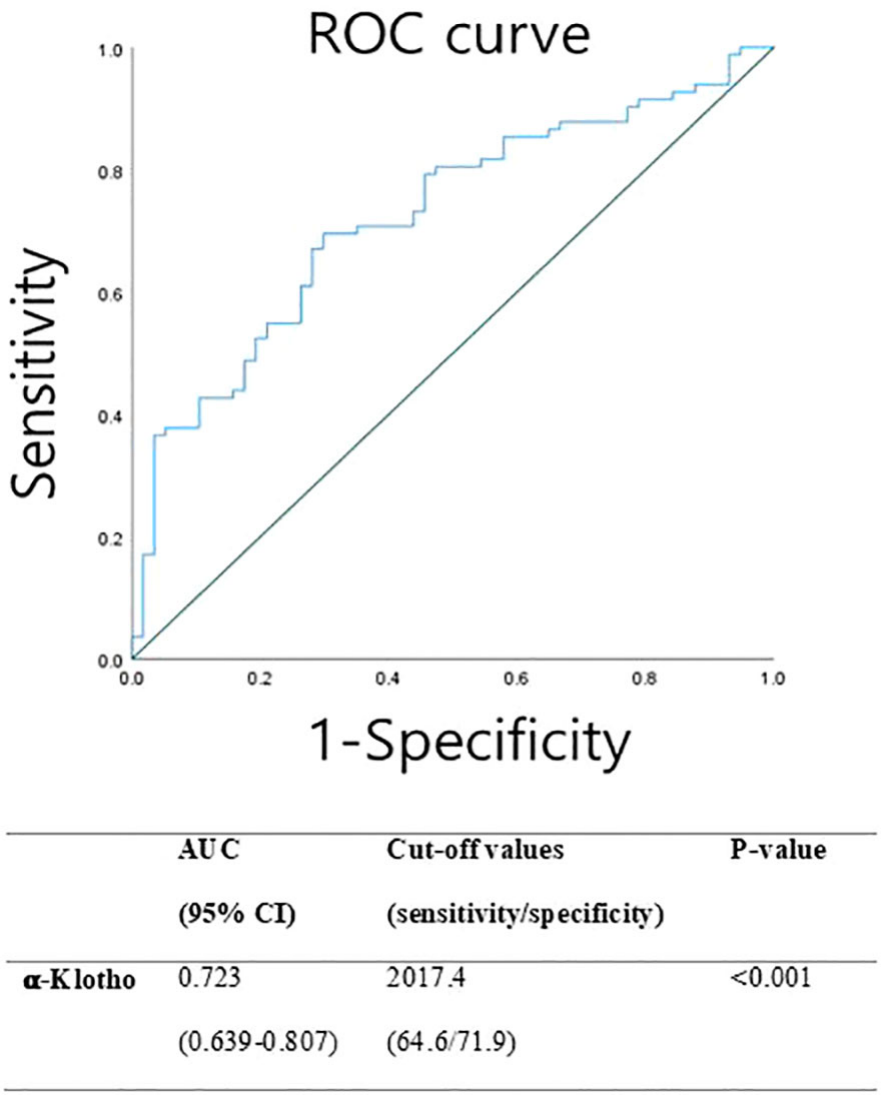
Receiver operating characteristic (ROC) curve representing the sensitivity and specificity of α-klotho for screening CPP.

ROC curve analysis was performed to assess the diagnostic performance of serum α-Klotho levels in discriminating between prepubertal control girls and girls with CPP (**Figure 1**). The optimal cut-off value for α-Klotho was determined to be 2017 pg/mL, yielding a sensitivity of 64% and specificity of 71%. The area under the curve (AUC) was 0.723, indicating the statistically significant diagnostic potential of α-Klotho in identifying CPP (p < 0.001).

**Figure 2:**
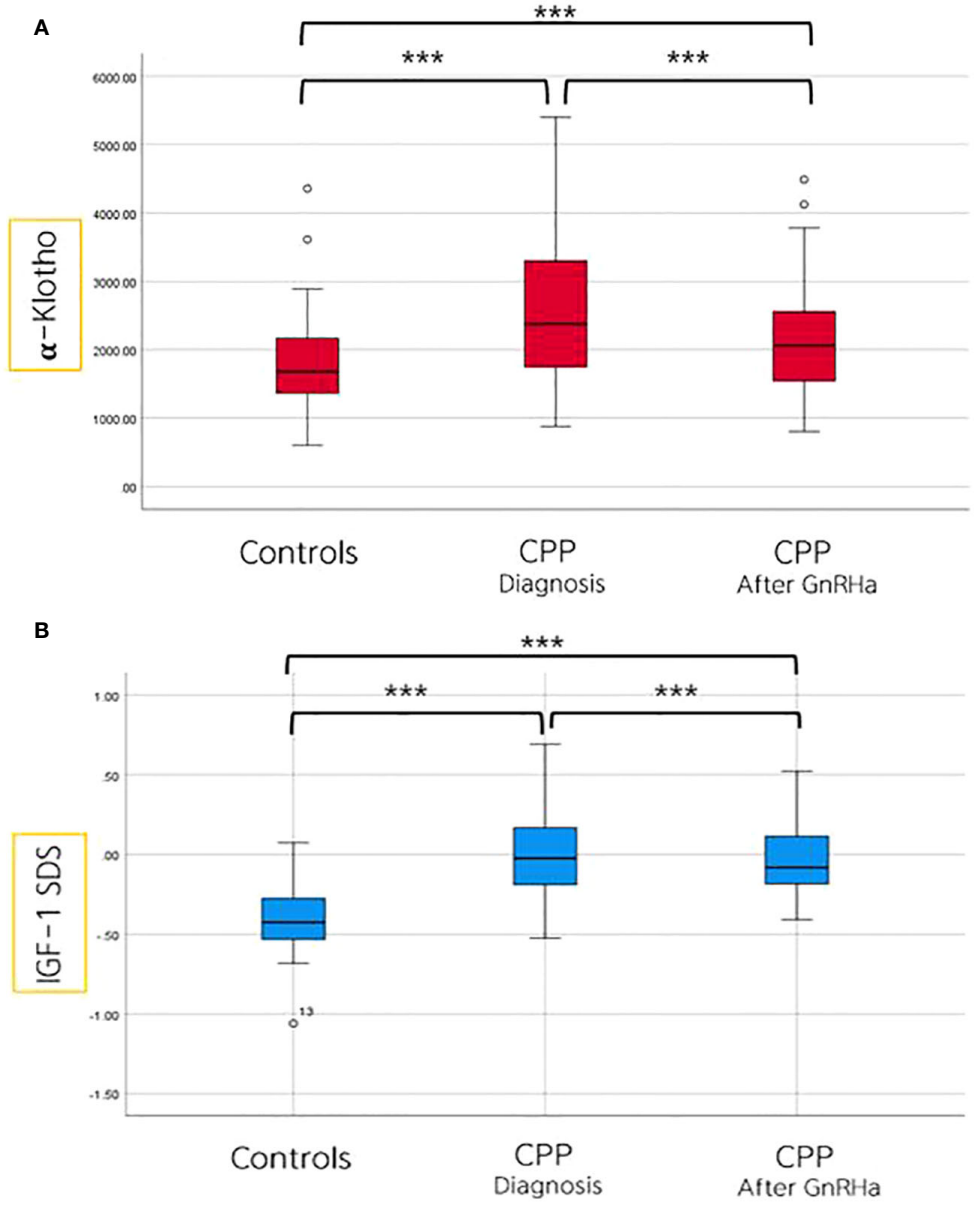
Comparison of α-Klotho **(A)** and IGF-I SDS **(B)** levels among controls, and in girls with CPP, both before and after 6 months of GnRHa treatment. ***p value ≤ 0.001.

After 6 months of GnRH agonist treatment in girls with CPP, significant reductions were observed in LH, estradiol, ALP, IGF-1 SDS, and α-Klotho levels. Particularly, α-Klotho levels decreased from an average of 2529 pg/mL before treatment to 2147 pg/mL after treatment (p = 0.001) (**Table 4**, **Figure 2**).

**Table 4:** Clinical, hormonal, and metabolic parameters of girls with CPP at diagnosis and after 6 months of GnRHa.

	Before treatment N = 82	6 month after treatment N = 82	P-value
**Age (years)**	8.42 ± 0.65	8.92 ± 0.65	NS
**BMI SDS**	0.30 ± 1.12	0.38 ± 1.03	NS
**Peak LH (mIU/mL)**	12.88 ± 8.65	0.58 ± 0.35	<0.001
**Peak FSH (mIU/mL)**	11.50 ± 3.72	2.41 ± 1.84	<0.001
**Estradiol (pg/mL)**	12.10 ± 12.00	5.09 ± 0.85	<0.001
**Calcium (mg/dL)**	9.74 ± 0.67	9.76 ± 0.61	NS
**Phosphate (mg/dL)**	4.89 ± 0.46	4.92 ± 0.46	NS
**ALP (IU/L)**	342.06 ± 69.29	266.52 ± 54.78	<0.001
**IGF-I SDS**	0.004 ± 0.26	-0.02 ± 0.24	0.002
**α-Klotho (pg/mL)**	2529.36 ± 999.51	2147.50 ± 789.62	<0.001

BMI, body mass index; CPP, central precocious puberty; N, number; NS, not significant; SDS, standard deviation score.

## 4 Discussion

Our findings demonstrated that girls with CPP exhibited significantly higher α-Klotho levels than prepubertal control girls in the same age group. Furthermore, we observed a modest decrease in α-Klotho levels in girls with CPP after 6 months of GnRH agonist treatment.

A previous study reported that children before and during puberty had median α-Klotho level of 2399 pg/mL (range, 762–5866 pg/mL), whereas the prepubertal group had a mean level of 1875 pg/mL (range, 372–5694 pg/mL) (14). In our study, the mean α-Klotho levels aligned with those findings, showing that girls with CPP had levels of 2529 ± 999 pg/mL and prepubertal girls had levels of 1802 ± 675 pg/mL. Our study specifically compared girls of the same age, enabling us to demonstrate a significant increase in α-klotho levels coinciding with the onset of puberty, thereby excluding the influence of aging on α-Klotho. To the best of our knowledge, this is the first investigation of α-Klotho levels in Korean girls, and we believe that continued data collection can establish these findings as valuable references for future medical research.

Our findings revealed that α-Klotho had a significant positive correlation with basal LH, FSH, and IGF-SDS, particularly after adjusting for age and BMI. This adjustment was performed because α-Klotho levels may be influenced by age and BMI (15–18). Previous studies have also supported the involvement of the insulin-like growth factor system in the initiation and progression of puberty, with documented positive correlations between α-Klotho and IGF-1 levels, indicating a closely regulated interplay between these factors (19–21). Specifically, it has been suggested that IGF-1 may stimulate Klotho secretion (22, 23). In addition, our multivariable logistic regression analysis for CPP revealed that α-Klotho is a significant factor influencing the outcome, similar to basal LH and estradiol. Furthermore, a previous study demonstrated the correlation between basal LH, FGF23, Klotho, and IGF-1 with rapidly progressive CPP, suggesting their potential importance in the rate of sexual development in girls (24). Building upon this body of literature and our own results, we hypothesized that an increase in LH, FSH, and IGF-1 levels during the onset of puberty may lead to higher α-Klotho levels. However, future large-scale studies with larger sample sizes are required to enhance the confidence in these associations.

Moreover, our study confirmed a modest decrease in α-Klotho levels 6 months after GnRH agonist treatment. The decline in levels, following the inhibitory treatment, suggests that elevated α-Klotho levels may be associated with hormonal changes in girls with CPP. Tracking α-Klotho levels may offer valuable insights for guiding potential future treatment directions and could serve as a promising monitoring marker. Through ongoing research efforts, we anticipate contributing to a better understanding of the pathological mechanisms of CPP and the development of more effective treatment strategies.

Nevertheless, this study had some limitations that warrant consideration. We were unable to assess α-Klotho levels in boys, and further investigation is required to determine whether similar patterns are observed in males. Additionally, the study did not evaluate α-Klotho levels after discontinuation of GnRH agonist treatment, which could provide insights into the long-term effects of treatment.

Our study sheds light on the intriguing role of α-Klotho in the context of CPP. Elevated α-Klotho levels in girls with CPP and their subsequent decrease following GnRH agonist treatment highlight its potential significance as a diagnostic and monitoring marker. The positive correlation between IGF-I and LH implies a complex interplay between hormones during the pubertal process, which warrants further investigation. While these findings are promising, additional research is required to fully elucidate the mechanisms underlying α-Klotho’s involvement in early puberty and its clinical utility as a diagnostic tool.

## Data availability statement

The original contributions presented in the study are included in the article/supplementary material. Further inquiries can be directed to the corresponding authors.

## Ethics statement

The studies involving humans were approved by Institutional Review Board of Kangdong Sacred Heart Hospital. The studies were conducted in accordance with the local legislation and institutional requirements. Written informed consent for participation in this study was provided by the participants’ legal guardians/next of kin.

## Author contributions

J-HP: Investigation, Methodology, Writing – original draft. E-SN: Conceptualization, Methodology, Writing – original draft. IH: Conceptualization, Writing – review & editing.
